# Supplementary material for: DDX5 plays essential transcriptional and post-transcriptional roles in the maintenance and function of spermatogonia
Source: Nat Commun. 2019 May 23;10:2278. doi: 10.1038/s41467-019-09972-7 (PMC6533336; doi:10.1038/s41467-019-09972-7)
Supplement: Supplementary file 1 — Supplementary Information [file 41467_2019_9972_MOESM1_ESM.pdf]

DDX5 plays essential transcriptional and post-transcriptional roles in the maintenance and function of spermatogonia

Legrand *et al*.

Supplementary Information

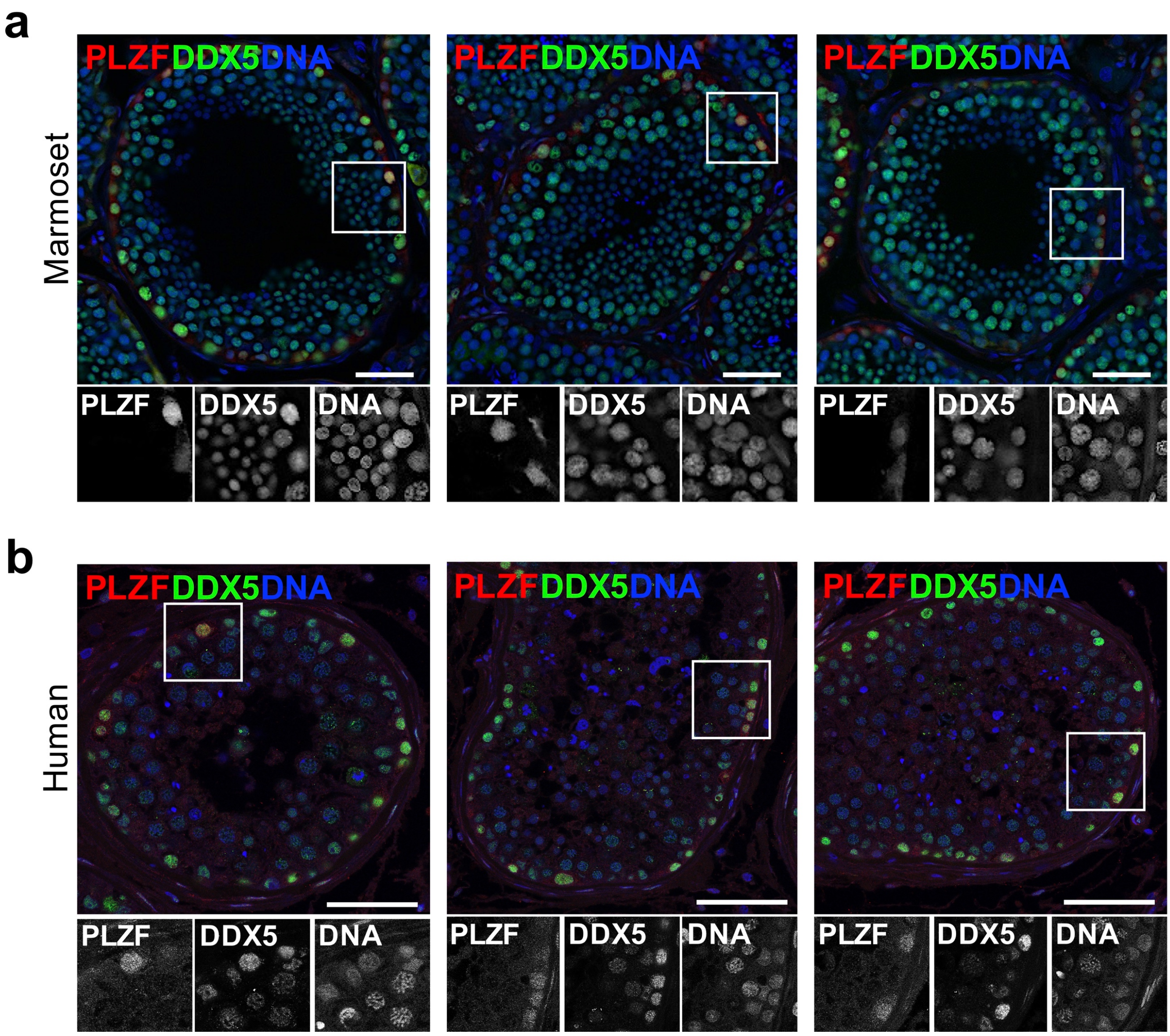

Supplementary Figure 1. DDX5 is expressed in non-human primate and human male germline cells. (**a**) Adult marmoset seminiferous tubule cross-sections showing nuclear expression of DDX5 in germ cells at all differentiation stages, including PLZF-positive spermatogonia. (**b**) Adult human seminiferous tubule cross-sections showing nuclear expression of DDX5 in germ cells at all differentiation stages, including PLZF-positive spermatogonia. All scale bars = 50μm.

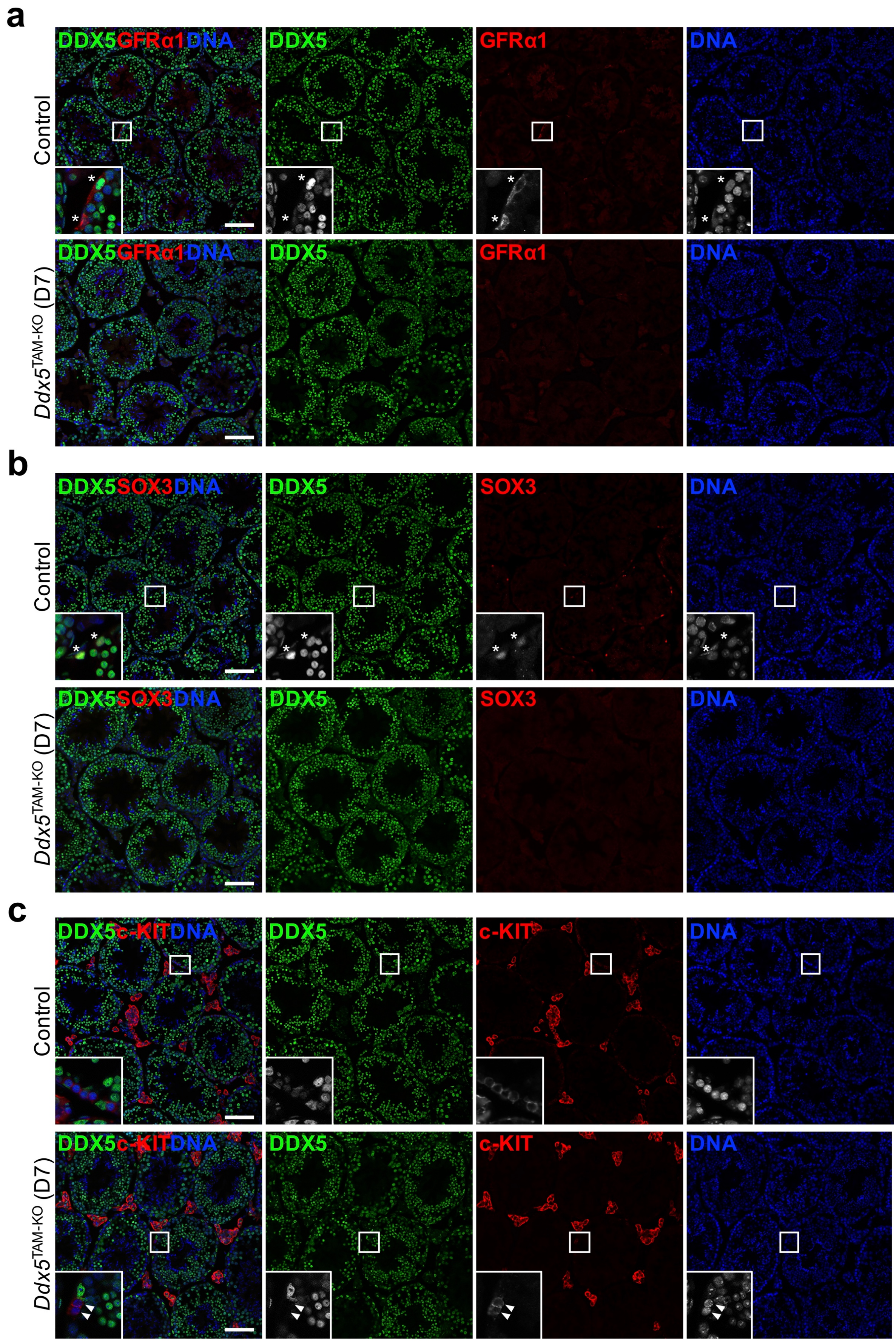

Supplementary Figure 2. *Ddx5* ablation *in vivo* results in loss of all spermatogonial subsets. Immunofluorescence of adult mouse seminiferous tubule cross-sections showing tamoxifen-induced UBC-Cre-mediated deletion of *Ddx5* (*Ddx5*$^{TAM-KO}$) results in loss of (**a**) GFRα1-positive, (**b**) SOX3-positive and (**c**) c-KIT-positive spermatogonia by 7 days post-tamoxifen (D7) compared to control mice. * in zoom insets show (a) GFRα1-positive, DDX5-positive spermatogonia and (b) SOX3-positive, DDX5-positive spermatogonia in control mice. Arrowheads in inset of (c) show few remaining c-KIT-positive, DDX5-negative spermatogonia remaining in *Ddx5*$^{TAM-KO}$ mice. All scale bars = 100μm.

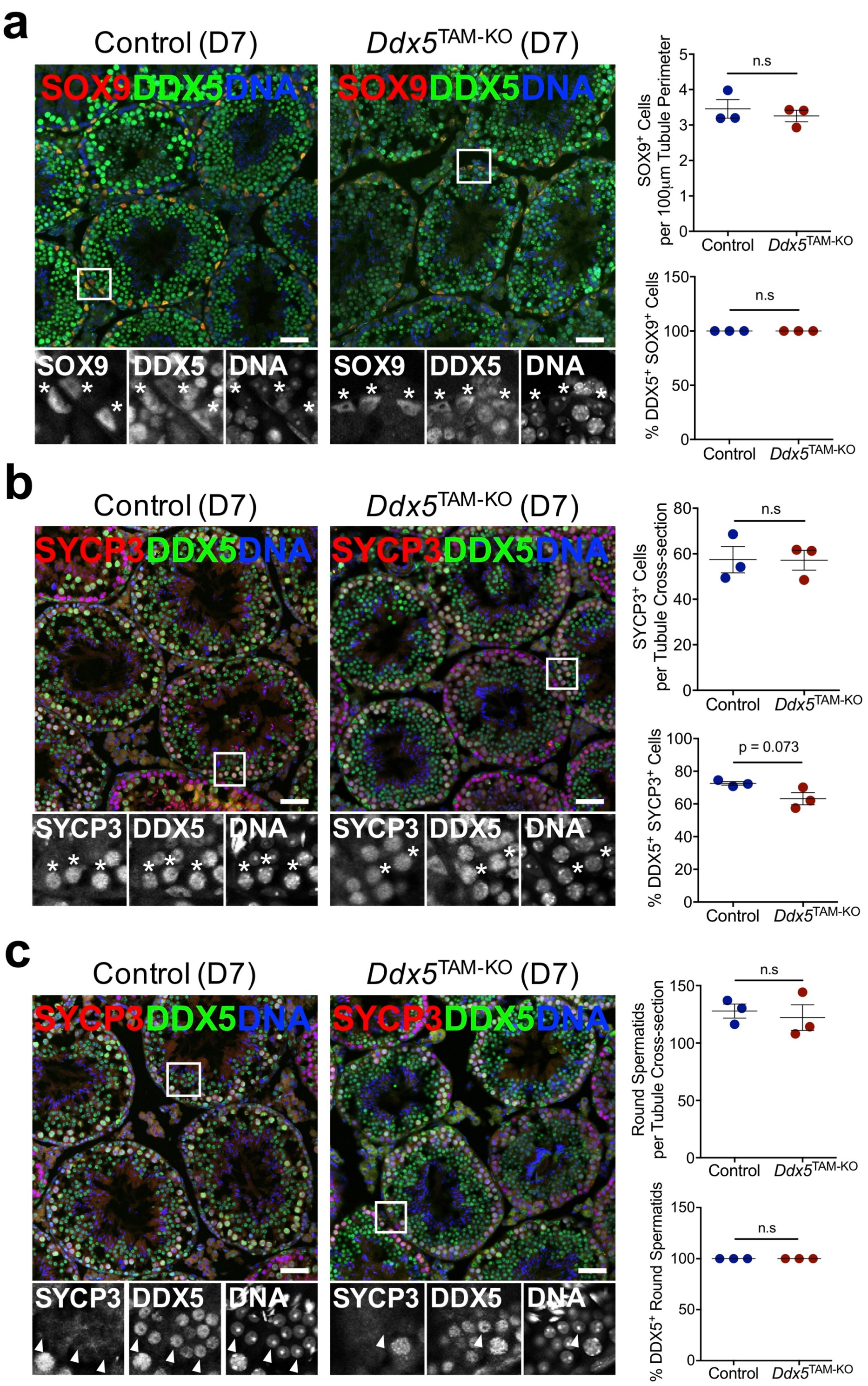

Supplementary Figure 3. Tamoxifen-induced *Ddx5* ablation in UBC-Cre^{ERT2}; *Ddx5*$^{flox/flox}$ mice does not affect Sertoli cells, spermatocytes or round spermatids. Immunofluorescence of adult mouse seminiferous tubule cross-sections showing tamoxifen-induced UBC-Cre-mediated deletion of *Ddx5* (*Ddx5*$^{TAM\text{-}KO}$) does not occur in (**a**) SOX9-positive Sertoli cells, (**b**) SYCP3-positive spermatocytes and (**c**) SYCP3-negative round spermatids at 7 days post-tamoxifen (D7). * in zoom windows show examples of (a) SOX9-positive, DDX5-positive Sertoli cells and (b) SYCP3-positive, DDX5-positive spermatocytes in control and *Ddx5*$^{TAM\text{-}KO}$ mice. Arrowheads in zoom windows of (c) show SYCP3-negative, DDX5-positive round spermatids with distinct morphology. All scale bars = 100µm. Quantification of cell numbers in right panels show (a) number of SOX9+ cells per 100µm tubule perimeter (top) and proportion of SOX9-positive cells that are DDX5-positive (bottom); (b) number of SYCP3-positive cells per tubule cross-section (top) and proportion of SYCP3-positive cells that are DDX5-positive (bottom); and (c) number of round spermatids per tubule cross-section (top) and proportion of round spermatids that are DDX5-positive (bottom) for control and *Ddx5*$^{TAM\text{-}KO}$ testes at D7 post-tamoxifen. n.s.: not significant (P>0.05); Mann-Whitney U test; mean±SEM; n=3 mice per condition.

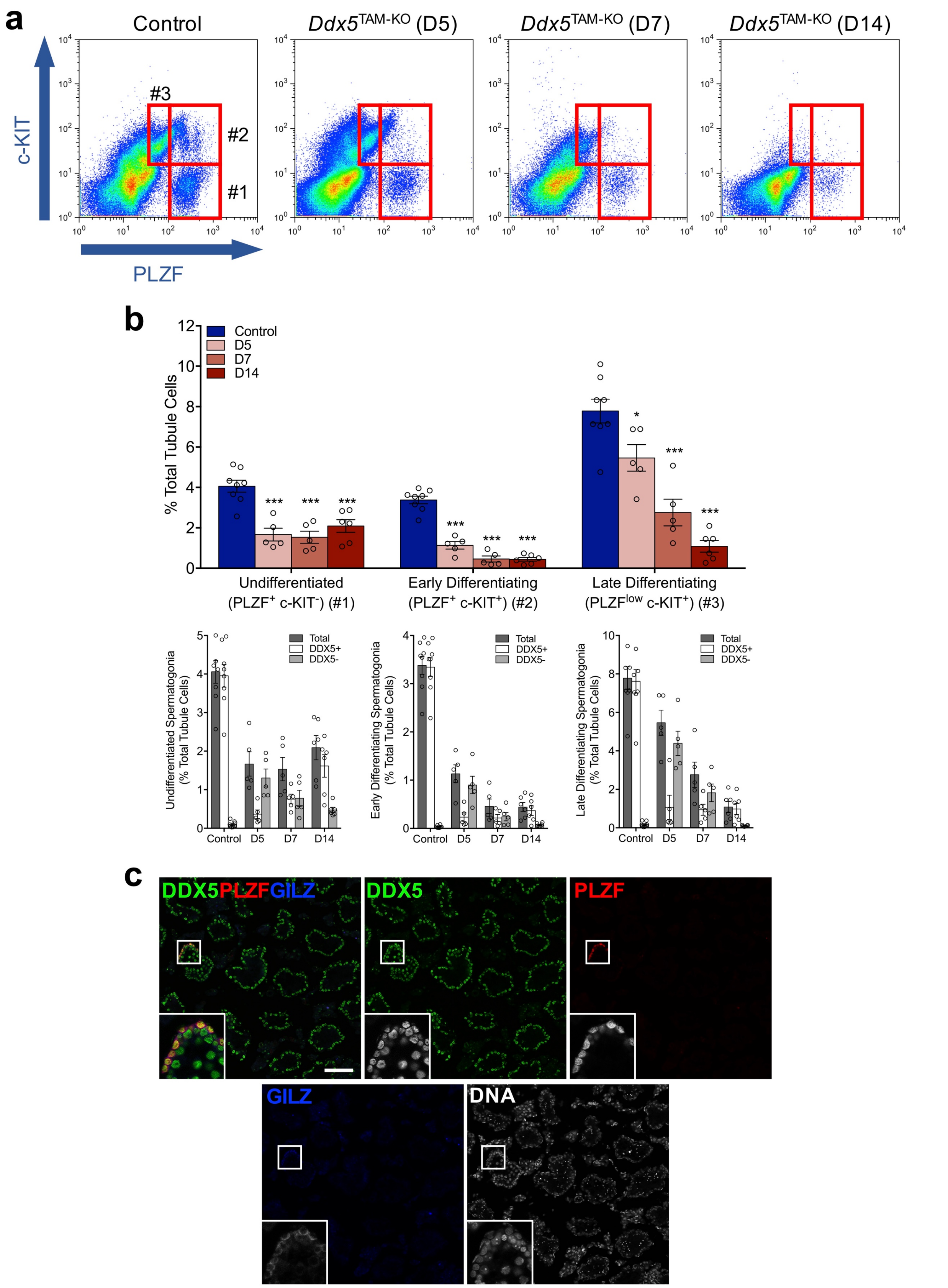

Supplementary Figure 4. *Ddx5*-ablation *in vivo* results in significant loss of all spermatogonia. (**a**) Representative flow cytometry plots from whole testis cell extracts of control versus *Ddx5*-ablated (*Ddx5*$^{\text{TAM-KO}}$) mice over time. Plots show PLZF^{+} c-KIT^{-} undifferentiated spermatogonia (#1); PLZF^{+} c-KIT^{+} early differentiating spermatogonia (#2); and PLZF^{low} c-KIT^{+} late differentiating spermatogonia (#3) populations at D5, D7 and D14 post-ablation. (**b**) Quantification of cell populations shown in **a**. *: $P<0.05$ versus control; ***: $P<0.001$ versus control; one-way ANOVA with Tukey multiple comparisons test; mean±SEM; n=8 control; n=5 D5, D7; n=6 D14. (**c**) Immunofluorescence of adult mouse seminiferous tubule cross-sections showing tamoxifen-induced UBC-Cre-mediated deletion of *Ddx5* (*Ddx5*$^{\text{TAM-KO}}$) results in Sertoli cell-only phenotype at D30. Zoom insets show small areas of *Ddx5*-retaining spermatogonia. Scale bar = 100µm.

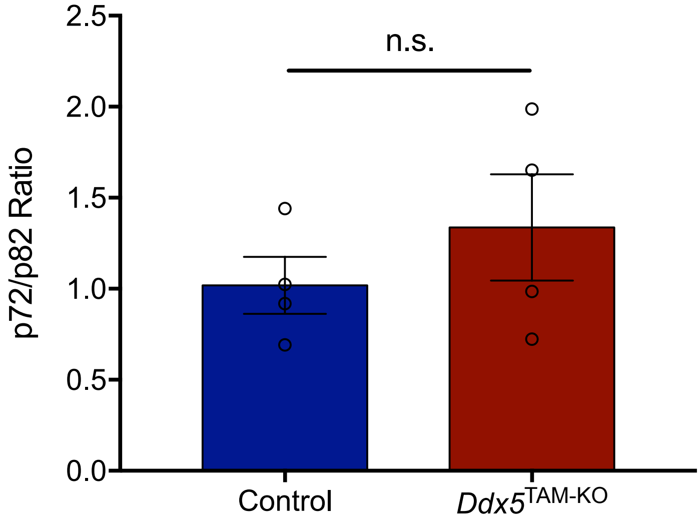

Supplementary Figure 5. *Ddx5* ablation does not affect ratio of DDX17 isoforms in spermatogonia. Quantification of DDX17 p72 and p82 isoforms in control and *Ddx5*-ablated (*Ddx5*$^{TAM-KO}$) spermatogonia (related to Figure 3c). Quantification was performed using ImageJ on western blots from n=4 independent replicates per condition. n.s.: not significant. Two-tailed unpaired t-test. Mean±SEM shown.

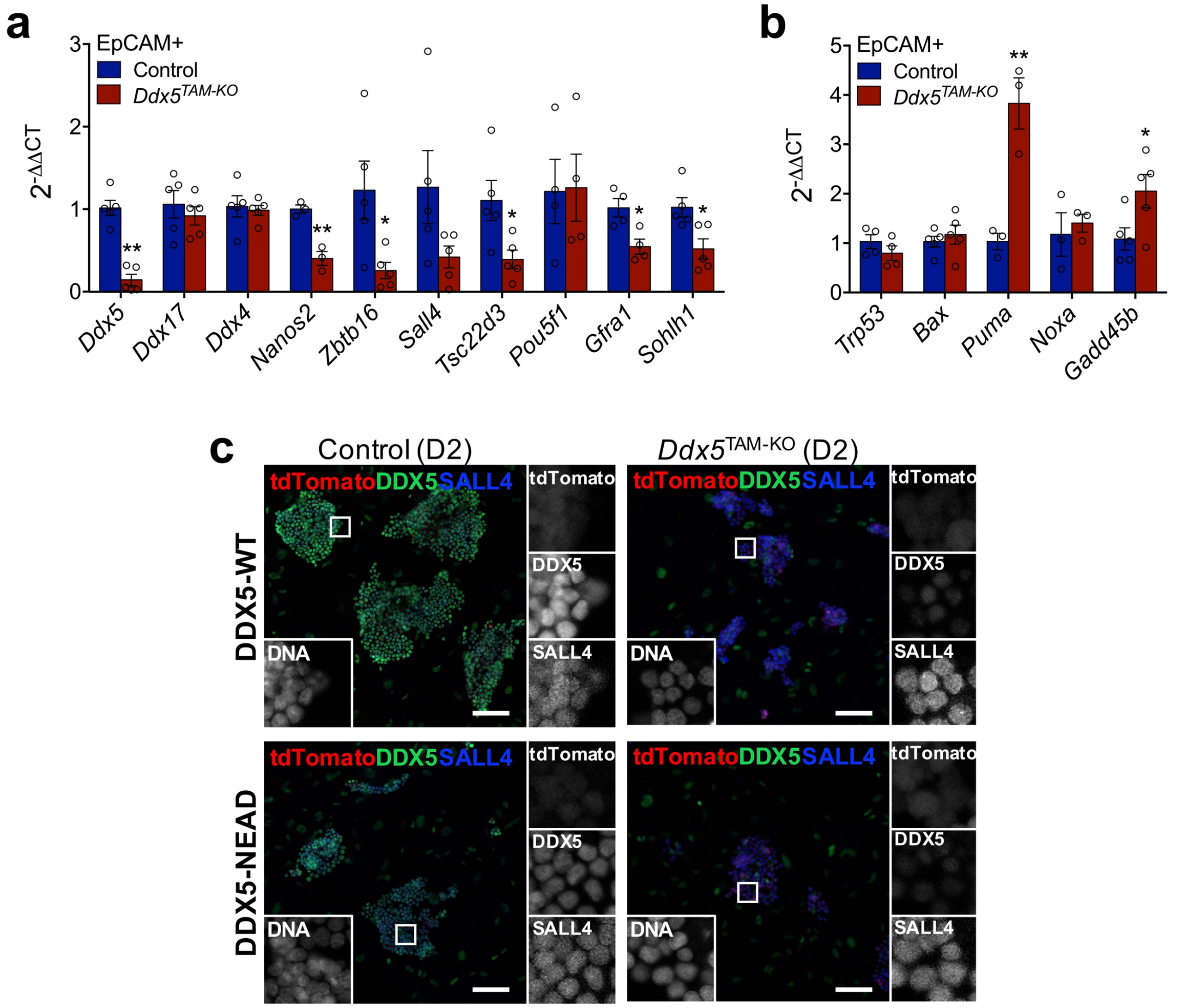

Supplementary Figure 6. RT-qPCR validation of RNA-seq and expression of DDX5 constructs *in vitro*. (**a**) RT-qPCR validation of RNA-sequencing data comparing control and *Ddx5*-ablated (*Ddx5*$^{\text{TAM-KO}}$) cultured undifferentiated spermatogonia (related to Figure 3d). Mean±SEM shown; *: P<0.05, **: P<0.01; Mann-Whitney U test for non-parametric data and two-tailed student's t-test for normally distributed data; min. n=3 independent cell lines per condition. (**b**) RT-qPCR validation of RNA-sequencing data comparing control and *Ddx5*-ablated (*Ddx5*$^{\text{TAM-KO}}$) cultured undifferentiated spermatogonia (related to Figure 3e). Mean±SEM shown; *: P<0.05, **: P<0.01; Two-tailed student's t-test; min. n=3 independent cell lines per condition. (**c**) Cultured UBC-Cre$^{\text{ERT2}}$*;Ddx5*$^{\text{flox/flox}}$ undifferentiated spermatogonia transduced with lentiviral DDX5 constructs tagged with tdTomato fluorescent protein. Constructs contained either wildtype DDX5 (DDX5-WT) or a helicase-inactive mutant of DDX5 (DDX5-NEAD). Cells were transduced, FACS-purified according to expression of tdTomato and replated on chamber slides prior to 4-hydroxytamoxifen-induced *Ddx5* ablation (*Ddx5*$^{\text{TAM-KO}}$). Treatment with vehicle only was used as a control. Cells were fixed at D2 post-ablation. SALL4 was used as a marker of spermatogonia. All scale bars = 50μm.

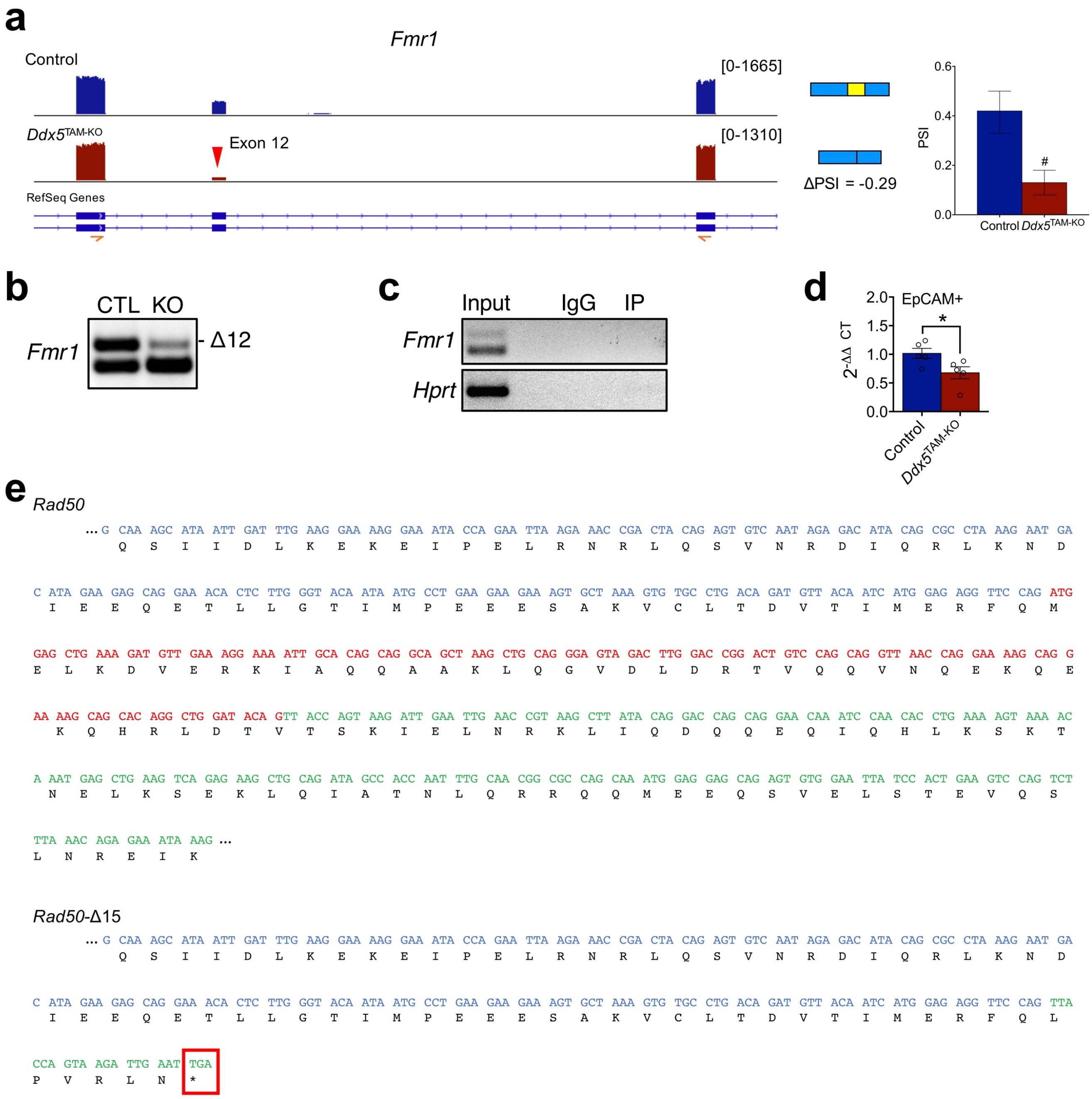

Supplementary Figure 7. DDX5 regulates splicing of *Rad50*. (**a**) Visualisation of differential splicing analysis of RNA-sequencing data comparing *Fmr1* in control and *Ddx5*-ablated spermatogonia (left). Red arrow indicates differentially spliced exon 12. Schematic of alternative splicing event is shown (blue and yellow rectangles) (middle). Change in "percent spliced in" between conditions is shown as a value below splicing schematic (ΔPSI) and in bar chart (right). #: changes meet analysis cut-offs (ΔPSI >0.20, Bayes Factor ≥10). PSI±95% confidence interval shown. (**b**) RNA-immunoprecipitation using DDX5 antibody in cultured wildtype spermatogonia followed by PCR and gel electrophoresis for *Fmr1* and negative control *Hprt*. (**c**) PCR validation of *Fmr1* differential splicing. CTL: vehicle-treated control spermatogonia; KO: *Ddx5*-ablated cultured spermatogonia. Δ12: isoform with excluded exon 12. Representative of n=3 independent samples. Position of the PCR primers used are depicted in (a) in orange beneath schematic showing gene structure. (**d**) RT-qPCR validation of RNA-sequencing data comparing *Rad50* expression in control and *Ddx5*-ablated (*Ddx5*$^{TAM\text{-}KO}$) cultured undifferentiated spermatogonia (related to Figure 4g). Mean±SEM shown; *: P<0.05; Two-tailed student's t-test; n=5 independent cell lines per condition. (**e**) Coding sequence analysis of *Rad50* showing exon 14 (blue), exon 15 (red) and exon 16 (green). Skipping of exon 15 (*Rad50*-Δ15) results in the introduction of a premature termination codon (indicated by red square).

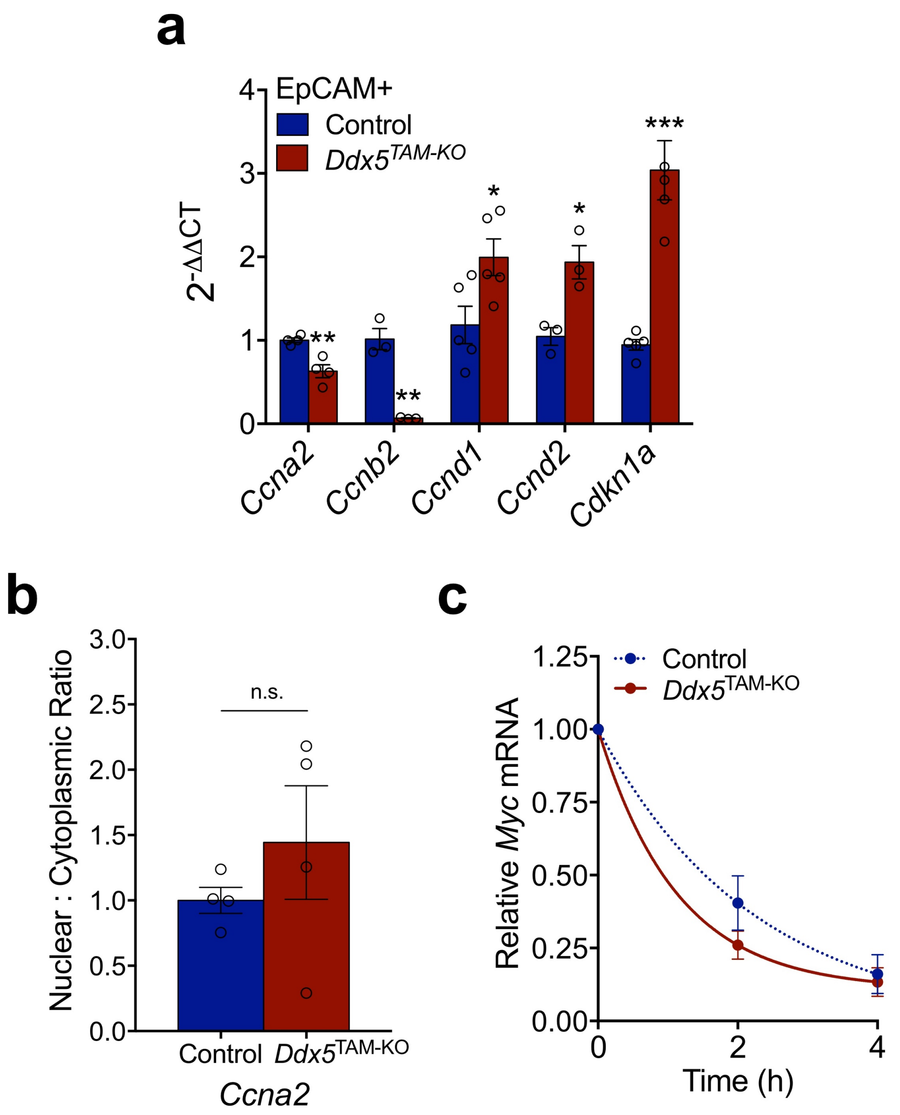

Supplementary Figure 8. DDX5 is a post-transcriptional regulator of specific mRNA transcripts in undifferentiated spermatogonia. (**a**) RT-qPCR validation of RNA-sequencing data comparing cell cycle-related gene expression in control and *Ddx5*-ablated (*Ddx5*$^{\text{TAM-KO}}$) cultured undifferentiated spermatogonia (related to Figure 5b). Mean±SEM shown; *: $P<0.05$, **: $P<0.01$, ***: $P<0.001$; Two-tailed student's t-test; n=5 independent cell lines per condition. (**b**) Nuclear-cytoplasmic ratio of *Ccna2* determined by RT-qPCR of control and *Ddx5*-ablated (*Ddx5*$^{\text{TAM-KO}}$) cultured undifferentiated spermatogonia subcellular fractions. Fold change relative to control shown. Mean±SEM shown; *: $P<0.05$; two-tailed unpaired t-test; n=4 per condition. (**c**) Analysis of *Myc* mRNA stability over time following inhibition of transcription with actinomycin D in control versus *Ddx5*-ablated (*Ddx5*$^{\text{TAM-KO}}$) cultured undifferentiated spermatogonia. mRNA expression determined by RT-qPCR (normalised to *Actb*) relative to initial levels at time 0h. Two-way ANOVA with Bonferroni multiple comparisons test; mean±SEM shown; n=4 per condition.

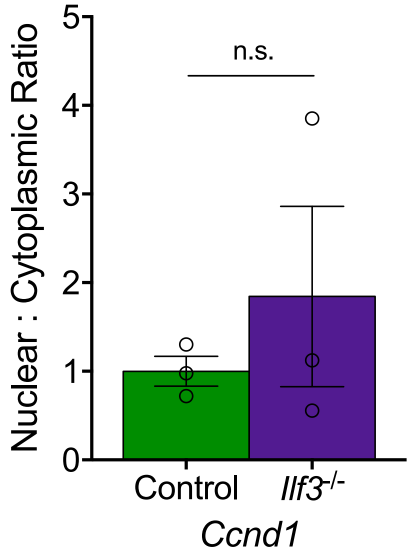

Supplementary Figure 9. Loss of *Ilf3* does not affect *Ccnd1* export in undifferentiated spermatogonia. Nuclear-cytoplasmic ratio of *Ccnd1* determined by RT-qPCR of control and *Ilf3*-ablated (*Ilf3*$^{-/-}$) cultured undifferentiated spermatogonia subcellular fractions. Fold change relative to control shown. Mean±SEM shown; n.s.: not significant (P>0.05); two-tailed unpaired t-test; n=3 per condition.

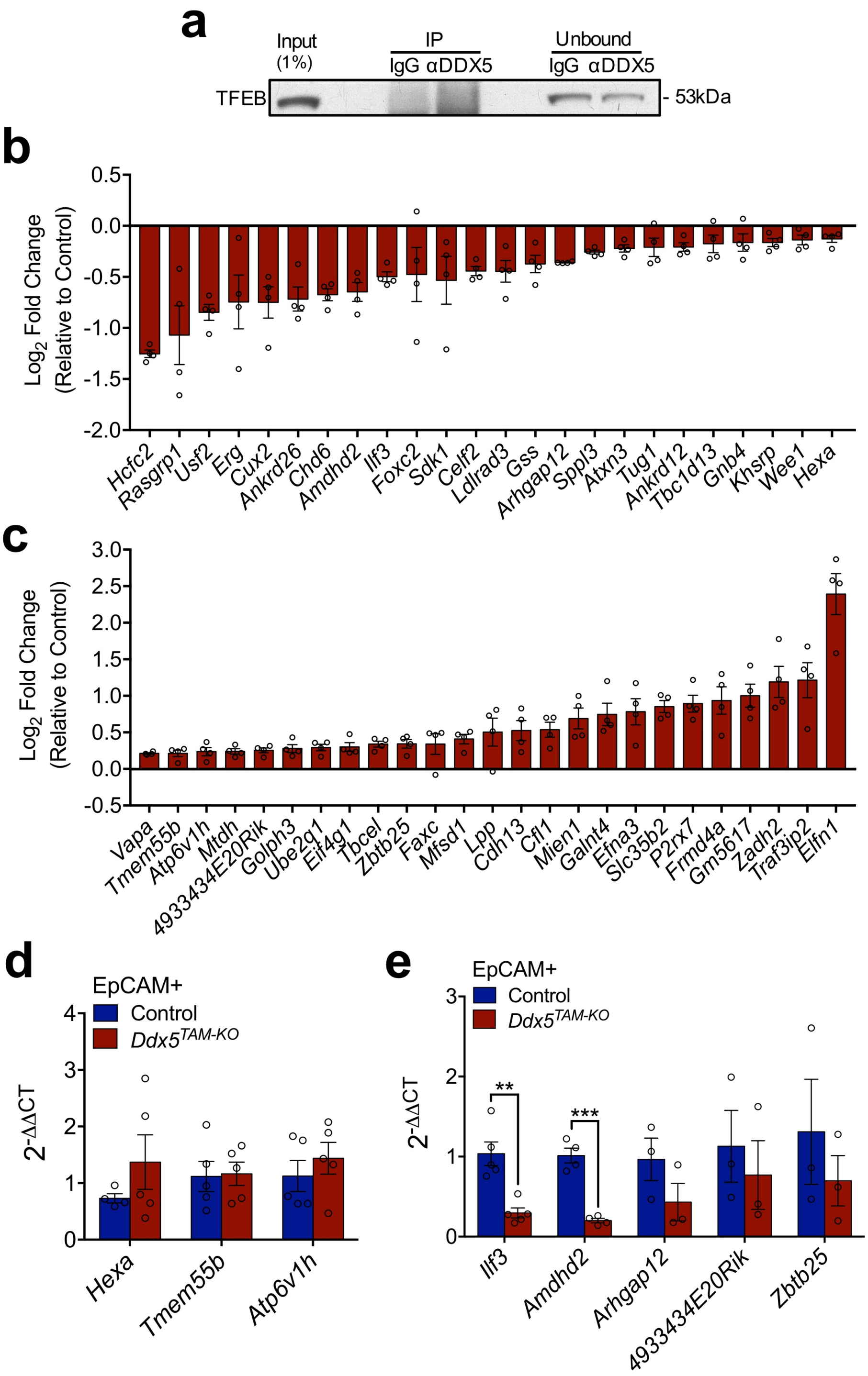

Supplementary Figure 10. Transcriptional regulation by DDX5 in undifferentiated spermatogonia. (**a**) DDX5 immunoprecipitation followed by western blot in wildtype cultured spermatogonia shows DDX5 does not interact with the autophagy regulator TFEB. Representative of n=2 independent experiments. (**b**) Downregulated genes identified by RNA-sequencing of control versus *Ddx5*-ablated cultured undifferentiated spermatogonia that are also bound by DDX5 according to ChIP-sequencing data. Fold change versus control shown. All differences meet FDR<0.05 cut-off. Mean±SEM shown; n=4 per condition. (**c**) Upregulated genes identified by RNA-sequencing of control versus *Ddx5*-ablated cultured undifferentiated spermatogonia that are also bound by DDX5 according to ChIP-sequencing data. Fold change versus control shown. All differences meet FDR<0.05 cut-off. Mean±SEM shown; n=4 per condition. (**d**) RT-qPCR validation of RNA-sequencing data comparing DDX5 autophagy-related targets in control and *Ddx5*-ablated (*Ddx5*$^{\mathrm{TAM\text{-}KO}}$) cultured undifferentiated spermatogonia (related to Figure 7d). Mean±SEM shown. All changes not significant. Two-tailed unpaired t-test. min. n=4 independent cell lines per condition. (**e**) RT-qPCR validation of RNA-sequencing data comparing DDX5-PLZF co-regulated candidate gene expression in control and *Ddx5*-ablated (*Ddx5*$^{\mathrm{TAM\text{-}KO}}$) cultured undifferentiated spermatogonia (related to Figure 7g). Mean±SEM shown; **: P<0.01, ***: P<0.001; Two-tailed unpaired t-test; min. n=3 independent cell lines per condition.

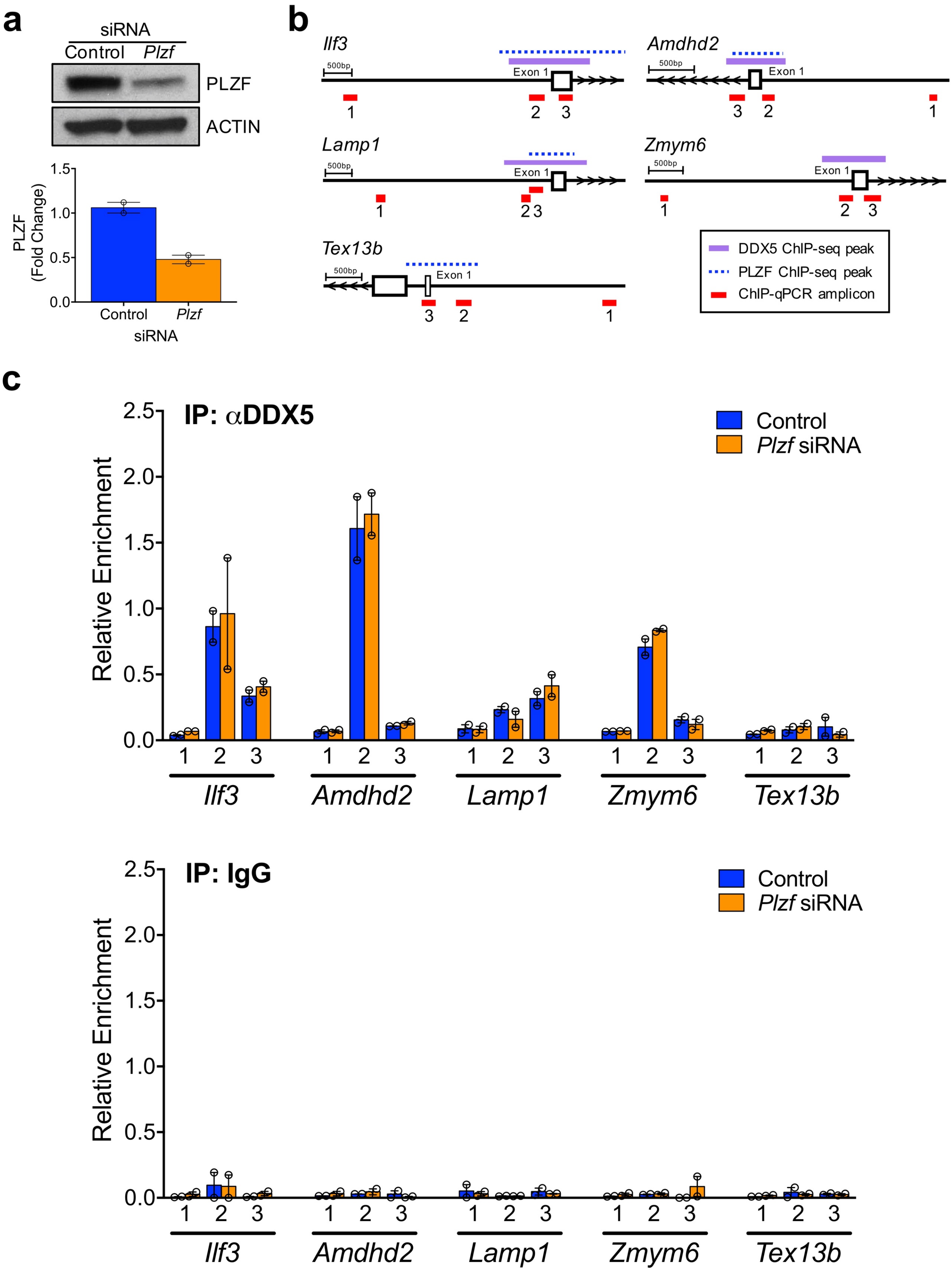

Supplementary Figure 11. Recruitment of DDX5 to gene promoters is not dependent on PLZF. (**a**) siRNA-mediated knockdown of *Plzf* in cultured undifferentiated spermatogonia. Representative western blot shown for PLZF following transfection with control non-targeting siRNA and *Plzf* siRNA pools (top panel). Quantification of western blots are shown in bottom panel (n=2 independent samples per condition). (**b**) Schematic of analysed genomic regions showing DDX5 (purple bar) and PLZF (blue dotted line) binding peaks determined by ChIP-seq. ChIP-qPCR amplicons are shown below (red bars) and are numbered 1 (upstream control region), 2 and 3 (within peaks). (**c**) DDX5 ChIP-qPCR in control and *Plzf* knockdown cultured spermatogonia. Numbers correspond to amplicons depicted in **b** for each gene. Data are presented as fold change relative to corresponding input sample (Relative Enrichment). ChIP with DDX5 antibody (top panel) and IgG negative control (bottom panel) are shown. n=2 independent samples per condition.

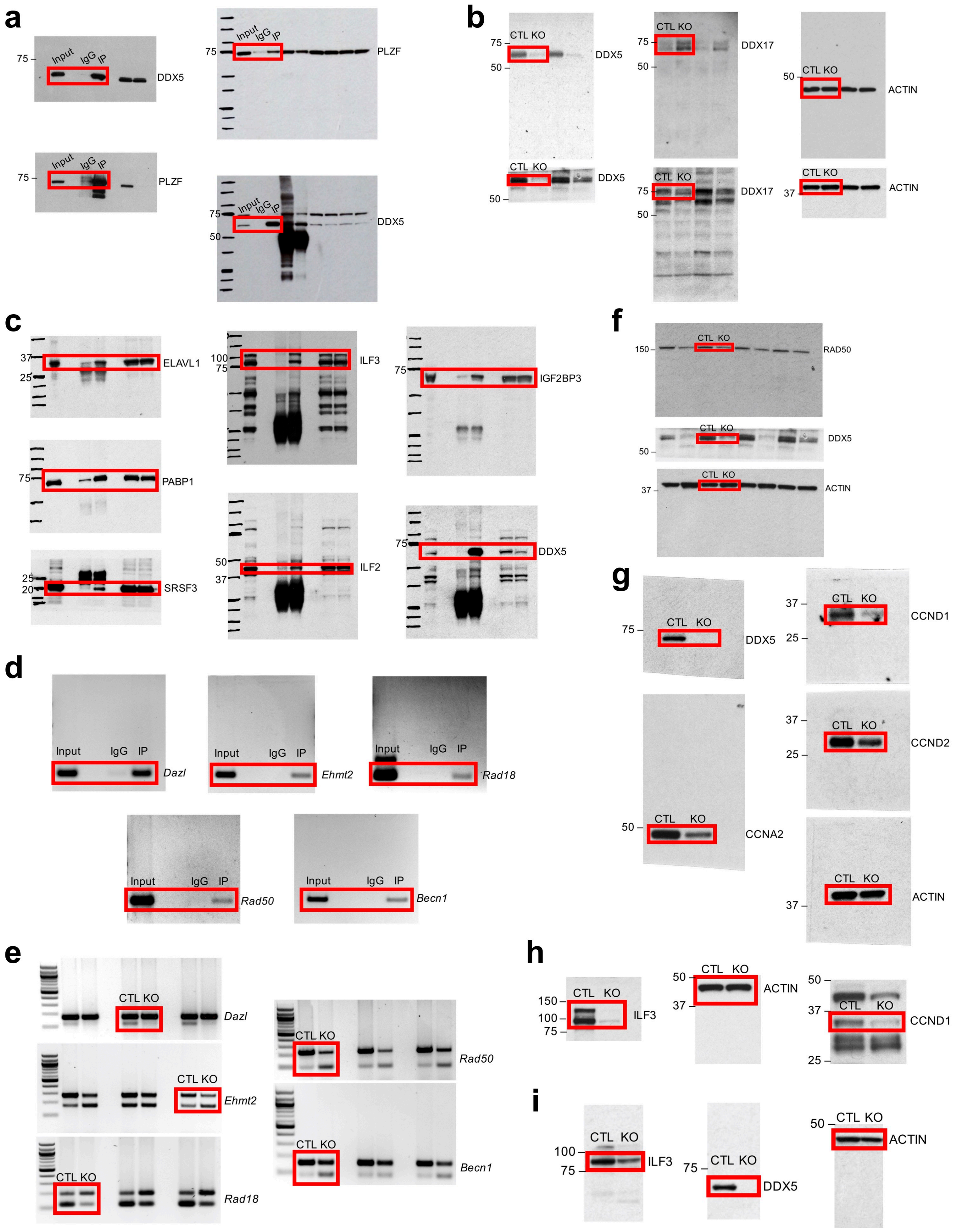

Supplementary Figure 12. Uncropped western blots and gels. Uncropped western blots and gels related to (**a**) Figure 1b; (**b)** Figure 3c; (**c**) Figure 4b; (**d**) Figure 4e; (**e**) Figure 4f; (**f**) Figure 4h; (**g**) Figure 7j5d; (**h**) Figure 6g; and (**i**) Figure 7j. Red boxes indicate area presented in main figures.

Supplementary Table 1. Primer sequences used for RT-PCR.

Gene	Forward (5'>3')	Reverse (5'>3')
Dazl (RIP-PCR)	TCCACCACAGTTCCAGAGTG	AACATAACTCCTCTGCTCTCCA
Dazl (Exon 8)	TCCTCCTTATCCAAGTTCACCA	TCAGCTCCTGGATCAACTTCAC
Ehmt2 (RIP-PCR)	CATCAATGCCGTAGATAAGCAA	GTGGAGCCATCCTCTTCCTT
Ehmt2 (Exon 10)	AGACAGCCCGTGGGTGAA	GCGGTCAATCTTGGGAGC
Rad50 (RIP-PCR/RT-qPCR)	TGATAAGTTGTCTTGGGGTTTCC	CTGTGTCTGACGCACCTGT
Rad50 (Exon 15)	GTGCTAAAGTGTGCCTGACA	CCTGCTGGTCCTGTATAAGC
Rad18	ACAAAGAACCAAACAGAGAAGGA	CTCTGCCAGTTTTCTTATACCCT
Becn1 (RIP-PCR)	CAGCTGGACACTCAGCTCAA	CGCTGGTACTGAGCTTCCTC
Becn1 (Exon 15)	ACTGGACACGAGCTTCAAGA	CCATCCTGGCGAGTTTCAAT
Ccna1	TGATGCTTGTCAAATGCTCAGC	AGGTCCTCCTGTACTGCTCAT
Ccna2	TGGATGGCAGTTTTGAATCACC	CCCTAAGGTACGTGTGAATGTC
Ccnb2	CAGTGACTACGTGAAGGACATC	TGGCACGCATACGTCCATTTA
Ccnd1	GTTCATTTCCAACCCACCC	CTCAGATGTCCACATCTCGC
Ccnd2	GATCACCCACACTGATGTGG	ATGACGAACACGCCTCTCTC
Ccnd3	CGAGCCTCCTACTTCCAGTG	GGACAGGTAGCGATCCAGGT
Ccne1	GTGGCTCCGACCTTTCAGTC	CACAGTCTTGTCAATCTTGGCA
Myc	GTCGCAGATGAAATAGGGCTG	ATGCCCCTCAACGTGAACTTC
Ilf3	GCAGGCTACAGTCAGTTCTACA	GGGGGTACTGGTGAGTTGT
Amdhd2	GATGTGCAGATCAACGGTGGA	CCTTGTGATAAACCTCTGGTGG
Arhgap12	GCTGGATGAACGTGGGCATA	ATTTCTCCTGACCCTTGTCGT
Tmem55b	TACGGAGCCGGTAAACATGC	GGCTAGTCAAGGGTGAGTAGG
433434E20Rik	CAAAGCACGAATTGGAAGTTCTC	GGTATGTGACCTGGATTGTTGTT
Zbtb25	TCAAGCAGGAGAGATGTGATCC	CCACAGTAATGGCATAAGTGGAC
Actin	GGCTGTATTCCCCTCCATCG	CCAGTTGGTAACAATGCCATGT
Ddx5	CGGGATCGAGGGTTTGGTG	GCAGCTCATCAAGATTCCACTTC
Ddx17	TCTTCAGCCAACAATCCCAATC	GGCTCTATCGGTTTCACTACG
Ddx4	TGTGCCACAACTTCTGAGGC	CCTGATTTCGGTTTCATCCATCC
Nanos2	CTGCAAGCACAATGGGGAGT	CGTCGGTAGAGAGACTGCTG
Zbtb16	CTCCGTAAGCGTCCCCTCTGC	GGTGCAGGCTAGCACCGTCC
Sall4	CCCTGGGAACCTGCGATGAAG	TCAGAGAGACTAAAGAACTCGGC
Pou5f1	CAGCCAGACCACCATCTGTC	GTCTCCGATTTGCATATCTCCTG
Tsc22d3	GGAGGTCCTAAAGGAGCAGATTC	GCGTCTTCAGGAGGGTGTTC
Gfra1	CACTCCTGGATTTGCTGATGT	AGTGTGCGGTACTTGGTGC
Sohlh1	GGCATCTGTCCTGGAGATGT	CACAGCAGATGGTTTGGGTA
Cdkn1a	CCTGGTGATGTCCGACCTG	CCATGAGCGCATCGCAATC
Ilf3	GCAGGCTACAGTCAGTTCTACA	GGGGGTACTGGTGAGTTGT
Amdhd2	GATGTGCAGATCAACGGTGGA	CCTTGTGATAAACCTCTGGTGG
Trp53	GTCACAGCACATGACGGAGG	TCTTCCAGATGCTCGGGATAC
Bax	TGAAGACAGGGGCCTTTTTG	AATTCGCCGGAGACACTCG
Puma	AGCAGCACTTAGAGTCGCC	CCTGGGTAAGGGGAGGAGT
Gadd45b	CAACGCGGTTCAGAAGATGC	GGTCCACATTCATCAGTTTGGC
Noxa	GCAGAGCTACCACCTGAGTTC	CTTTTGCGACTTCCCAGGCA
Hprt	GCTGGTGAAAAGGACCTCT	CACAGGACTAGAACACCTGC

Supplementary Table 2. Primer sequences used for ChIP-qPCR.

**Gene**	**Forward (5'>3')**	**Reverse (5'>3')**
Ilf3 (1)	CACTCAGGGGCTACGAAGAA	CATCTCCCTCTGCATCTGGA
Ilf3 (2)	CGTCAGGTGATTCGGGTCTA	GACTTAGCGACTGGGTCTGT
Ilf3 (3)	TTGCAGTTGGGACTGAAGCA	GACGCCACATTGCCACTTAC
Amdhd2 (1)	CCAAGTATACGGGAAGGTGGT	TCTCCCCTAGTCCTGTCGTG
Amdhd2 (2)	GTCGCATCACGTGACTCTCA	AGTTCTCGGATGTGACTGGC
Amdhd2 (3)	CTCCCTGAGGGCAGAGTAGT	CATAAGCCCTGAAAGGGGCA
Lamp1 (1)	TTCCTTGTGAGAAGACGCCT	TTCAAATCCCAGGACCCACA
Lamp1 (2)	TTCCACATCTCCCACCATCT	ATTCCCATCTAGACCCGTGC
Lamp1 (3)	GCACGGGTCTAGATGGGAAT	AAGGGAAGGAGAAACGCTGT
Zmym6 (1)	TTGAGGCAAAAGTAGGCAGG	GAAGATAAGCCTCTACTGTGGG
Zmym6 (2)	GCTTTTGTGCATTCTCCGAC	ACGTGAGGTTACCCAGAACT
Zmym6 (3)	GCTTGCGACATTCAGAGGAG	AATGGCTGCTAGAAACCCCT
Tex13b (1)	GAAGGAAGGCAGAGACAGGT	GTGTGTGTGTGTGACTGTGG
Tex13b (2)	CTCAAACTCCAGCCACACAG	TGGAAGACAGGAAAGGGACC
Tex13b (3)	TGAAGGCTAGCGACCAAAGA	TTCCGATGAGACTTGGCCTT
