# Supplementary material for: DDX5 plays essential transcriptional and post-transcriptional roles in the maintenance and function of spermatogonia
Source: Nat Commun. 2019 May 23;10:2278. doi: 10.1038/s41467-019-09972-7 (PMC6533336; doi:10.1038/s41467-019-09972-7)
Supplement: Supplementary file 3 — Description of Additional Supplementary Files [file 41467_2019_9972_MOESM3_ESM.pdf]

Description of Additional Supplementary Files

File Name: Supplementary Data 1
Description: PLZF IP-Mass Spectrometry Identified Proteins. Eluted proteins identified by mass spectrometry following co-immunoprecipitation with PLZF antibody from cultured undifferentiated spermatogonia lysates

File Name: Supplementary Data 2
Description: RNA-sequencing – Control vs. *Ddx5* KO. Differentially expressed genes identified by RNA-sequencing of vehicle-treated control versus *Ddx5*-ablated cultured undifferentiated spermatogonia

File Name: Supplementary Data 3
Description: DDX5 IP-Mass Spectrometry Identified Proteins. Eluted proteins identified by mass spectrometry following co-immunoprecipitation with DDX5 antibody from cultured undifferentiated spermatogonia lysates

File Name: Supplementary Data 4
Description: Gene Ontology Analysis (Molecular Function) by PANTHER of DDX5 IP-Mass Spectrometry Identified Proteins. Gene ontology analysis for enriched GO molecular function terms using data from DDX5 IP-mass spectrometry experiments

File Name: Supplementary Data 5
Description: Gene Ontology Analysis (Biological Process) by PANTHER of DDX5 IP-Mass Spectrometry Identified Proteins. Gene ontology analysis for enriched GO biological function terms using data from DDX5 IP-mass spectrometry experiments

File Name: Supplementary Data 6
Description: Differentially Splicing Analysis by MISO (Control vs. *Ddx5* KO RNAseq). Differential splicing analysis by MISO using RNA-sequencing data obtained from vehicletreated control versus *Ddx5*-ablated cultured undifferentiated spermatogonia

File Name: Supplementary Data 7
Description: Ingenuity Pathway Analysis of Control vs. *Ddx5* KO Differentially Expressed Genes (by RNA-seq). Pathway analysis of differentially expressed genes identified by RNA-sequencing of vehicle-treated control versus *Ddx5*-ablated cultured undifferentiated spermatogonia using the Ingenuity platform

File Name: Supplementary Data 8
Description: DDX5 ChIP-sequencing. Peaks identified by HOMER following DDX5 ChIP-sequencing in cultured undifferentiated spermatogonia
